# Association of *rs4784227-CASC16 (LOC643714 locus)* and *rs4782447-ACSF3* polymorphisms and their association with breast cancer risk among Iranian population

**DOI:** 10.17179/excli2019-1374

**Published:** 2019-06-18

**Authors:** Amir Tajbakhsh, Zahra Farjami, Susan Darroudi, Seyed Hasan Ayati, Fatemeh Vakili, Mahla Asghari, Maliheh Alimardani, Soheila Abedini, Mohammad Mahdi Kushyar, Alireza Pasdar

**Affiliations:** 1 Department of Modern Sciences & Technologies, Faculty of Medicine, Mashhad University of Medical Sciences, Mashhad, Iran; 2 Young Researchers and Elite Club, Yasooj Branch, Islamic Azad University, Yasooj, Iran; 3 Student Research Committee, Faculty of Medicine, Mashhad University of Medical Sciences, Mashhad, Iran; 4 Immunology Research Center, Department of Immunology, Medical School, Mashhad University of Medical Sciences. Mashhad, Iran; 5 Midwifery department, Faculty of Nursing and Midwifery, Mashhad University of Medical Sciences, Mashhad, Iran; 6 Department of Medical Genetics, Faculty of Medicine, Mashhad University of Medical Sciences, Mashhad, Iran; 7 Department of Haematology-Oncology, Imam Reza Hospital, Mashhad University of Medical Sciences, Mashhad, Iran; 8 Division of Applied Medicine, Faculty of Medicine, University of Aberdeen, Foresterhill, Aberdeen, UK

**Keywords:** chromatin remodelling, carcinoma, genetic variation, epidemiology, enhancer element

## Abstract

TOX3 and FOXA1 proteins are believed to be involved in the susceptibility of breast cancer. *rs4784227-CASC16* and* rs4782447-ACSF3*, as single nucleotide polymorphisms (SNPs), located at the *16q* may affect the FOXA1 DNA binding sequence change and therefore may enhance the FOXA1-binding affinity to the promoter of *TOX3* gene. This study aimed to investigate the association of these SNPs/haplotypes with breast cancer susceptibility in an Iranian population. We conducted a case-control study of 1072 blood samples (505 breast cancer patients and 567 controls). Genotyping of *rs4784227-CASC16* and *rs4782447-ACSF3* SNPs was carried out by ARMS-PCR. Moreover, statistical analysis was done using SPSS version 20.0 (IBM Inc., Chicago, IL, USA), PHASE v 2.1 and SNP analyser 2.0. There was a strongly significant statistical association between alleles and genotypes of *rs4784227-CASC16* with breast cancer risk in our study population (p<0.05). Moreover, a significant association was demonstrated between TA haplotype and breast cancer risk (OR=0.78; 95% CI (0.62-0.96); P-*_value_*=0.025). In this respect, although we did not observe a statistically significant association between *rs4782447-ACSF3* with breast cancer susceptibility, the combination of the effects of *rs4784227-CASC16* and *rs4782447-ACSF3* SNPs may also affect the risk. This is in line with other studies suggesting these SNPs as risk-associated polymorphisms which may lead to a change in the affinity of FOXA1, as a distal enhancer, to *TOX3* and thus change in *TOX3* expression, which can eventually affect the risk of breast cancer.

## Introduction

Based on the previous studies, using genome-wide association studies (GWASs), 72 susceptibility regions of breast tumour have been found (Ghoussaini et al., 2013[11]). Numerous genes near the identified susceptibility loci have genes with unknown function, such as *16q12* locus which encompasses *TOX3*/ *LOC643714* gene (Ghoussaini et al., 2013[11]). TOX3 clinical implications and its role in tumour development and the invasion have been shown in the risk of breast cancer (Chalabi et al., 2008[6]; Mahfoudh et al., 2012[21]; Tajbakhsh et al., 2017[30], 2019[31]). Generally, TOX3 is introduced as a member of the high-mobility-group (HMG) family of proteins that modifies chromatin structure (O'Flaherty and Kaye, 2003[23]). Change of *TOX3* expression is associated with expression of progesterone receptor (PR) and oestrogen receptor (ER) and also positive lymph nodes (Gudmundsdottir et al., 2012[12]). In this line, it is indicated that low level of *TOX3* expression has been correlated with high level of Ki67 and also the subtype of basal tumour while high mRNA expression was connected with ER positive, PR positive, and positive lymph nodes in the tumour and normal tissue samples (Gudmundsdottir et al., 2012[12]). Interestingly, *TOX3/LOC643714* is related to ER^- ^or ER^+^ of breast cancer subtypes (Ghoussaini et al., 2013[11]). In this regard, numerous single nucleotide polymorphisms (SNPs), located in the DNA binding site, which are bound by FOXA1, are connected with the risk of breast cancer (Lupien et al., 2008[20]). It has been shown that FOXA1 has a key role in the function of ER and growth of ER^+^ cells of breast cancer (Carroll et al., 2005[5]; Kong et al., 2011[18]). Importantly, many breast cancer risk-associated SNPs can affect FOXA1-binding affinity for enhancer sequences and eventually increase or prevent transcriptional activity of ER (Meyer and Carroll, 2012[22]). The co-localization of *TOX3* with FOXA1 is notable as *TOX3* expression may be regulated by FOXA1 (Bernardo and Keri, 2012[3]; Bernardo et al., 2010[4]). It is suggested that FOXA1, through binding to an upstream enhancer, can be a positive regulator for the *TOX3* expression (Cowper-Sal·lari et al., 2012[8]). A TOX3-FOXA1 interaction might have a role throughout the differentiation of progenitor ER^-^ positive luminal cell type in normal cells (Cowper-Sal·lari et al., 2012[8]; Seksenyan, 2013[26]). 

**Figure 1:**
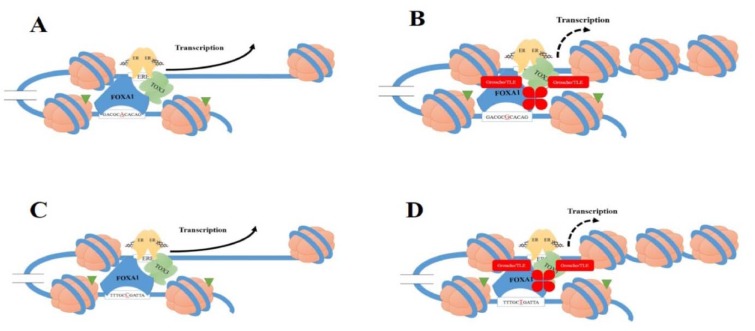
Schematic figure of two single nucleotide polymorphisms (SNPs), rs4782447 and rs4784227, located at the *16q* are indicated that lead to the forkhead-box A1 (FOXA1) binding sequence change and therefore may increase the affinity of FOXA1 and/or Groucho (Gro)/transducin-like enhancer of split (TLE) proteins binding to the promoter of TOX3 gene. A: A sequence with wild type allele (A allele) of rs4782447; B: A sequence with mutant allele (G allele) of rs4782447; C: A sequence with wild type allele (C allele) of rs4784227; D: A sequence with mutant allele (T allele) of rs4784227. A and C: parts of this figure show an increase of transcription, but part B and D indicate a decrease of transcription. Based on the previous studies, sequences that carrier mutant allele increase affinity of FOXA1 and/or Gro/TLE to bind to TOX3. Co-bonding of FOXA1 and Gro/TLE may reduce transcription of TOX3.

There are several important non-coding SNPs related to *TOX3/LOC643714 *locus that may change the affinity of FOXA1 to *TOX3*. Moreover, the Encyclopedia of DNA Elements (ENCODE) is indicating about 80 % of the non-coding DNA may be functional (The Encode Project Consortium, 2012[32]). In breast cancer cells, the disease risk allele of non-coding SNP enhances the FOXA1-binding affinity for the upstream enhancer of *TOX3* gene which in turn can change *TOX3* expression (Cowper-Sal·lari et al., 2012[8]) (Figure 1(Fig. 1)). The growing evidence indicates that *16q12.1* locus, which harbour *rs4784227-CASC16* SNP, has been connected with breast tumour in GWASs in European, Asian and African ancestry populations (Easton et al., 2007[9]; Long et al., 2010[19]; Ruiz-Narvaez et al., 2010[25]; Stacey et al., 2007[28]; Udler et al., 2010[33]). Moreover, *rs4782447-ACSF3* is also reported to play a significant role in the risk of breast cancer (Meyer and Carroll, 2012[22]). The interpretation of genetic connections between pathogenesis of breast cancer and SNPs and/or haplotypes have been extremely investigated (Yoo et al., 2008[36]). Understanding genetic variations may help understand the biological mechanisms of development, progression, inhibition, early diagnosis and the tailored treatment of the disease (Barrdahl et al., 2015[2]). No study has been done in the association between these important SNPs and the haplotypes with breast cancer risk among Iranian population. Thus, in this article, we tried to investigate the association between *16q* region including *TOX3/LOC643714 locus*, which interacts with FOXA1, and breast cancer risk in a cohort of Iranian population.

## Materials and Methods

### Study population and clinical data

Following approval by the ethics committee of Mashhad University of Medical Sciences (IR.MUMS.fm.REC.1394.399), 1072 blood specimens were collected from 567 healthy controls and 505 patients. A written informed consent form was signed by all individuals. A questionnaire was used to collect demographic information. 

### Blood collection and DNA extraction

10 ml of whole peripheral blood was collected from each individual and divided into tubes having sterile ethylene diamine tetra acetic acid (EDTA) for DNA extraction. DNA extraction was completed using salting out technique and was quantified at a wavelength of 260 nm and 280 nm through BioTek™ Epoch™ Microplate Spectrophotometer (Winooski, VT, USA,) and also by gel electrophoresis. 

### Target SNPs determinations (Marker selection)

In the present study, target SNPs were determined using available SNP public databases, and also related published articles. These articles have investigated non-coding SNPs that may change the affinity of FOXA1 to *TOX3/LOC643714* in breast cancer. Moreover, we tried to select SNPs that are not located in strong linkage disequilibrium (LD) to prevent redundancy in genotyping.

### Genotyping 

To determine the genotype frequency of *rs4782447-ACSF3* and *rs4784227-CASC16* SNPs, ARMS-PCR was used. PCR amplifications for *rs4782447-ACSF3* and *rs4784227-CASC16* have been carried out in a 10 μl final volume per reaction containing three µl Taq 2x master mix (Ampliqon, Germany), one µl of each primer (10 µM) and 100 ng DNA. The primers used for detection of *rs4782447-ACSF3 *and *rs4784227-CASC16* SNPs are listed in Table 1(Tab. 1). The ARMS-PCR condition for rs4782477 was as follows: initial denaturation at 94 °C for five minutes, after that 35 cycles including denaturation at 94 °C for 25 seconds, annealing at 59 °C for 25 seconds, an extension at 72 °C for 30 seconds followed by 72 °C for seven minutes as the final extension step. Moreover, ARMS-PCR condition for *rs4784227-CASC16* was the same as rs4782477 with a different annealing temperature of 71 °C. The DNA fragments of PCR products were detected using electrophoresis in 2 % agarose gel.

### Statistical analysis

Hardy-Weinberg equilibrium (HWE) assumption was investigated using the Pearson χ^2^ distribution. The association between breast cancer, risk factors and alleles/genotypes were assessed using binary logistic regression, which estimated Odds ratios (ORs) as well as 95% confidence intervals (CIs). For all analyses, a P-*_value_*=0<0.05 was considered statistically significant. Logistic regression was also used to measure the associations of risk factors using different genetic models. SPSS 20.0 (Inc., Chicago, IL, USA) and also SNP analyser 2 software (Yoo et al., 2008[36]) were used for statistical analysis.

### Haplotype analysis

Haplotypes were assembled from genotype data using PHASE program and SNP analyser 2 software (Stephens et al., 2001[29]; Yoo et al., 2008[36]). In this study, P-*_values_* less than 0.05 were considered as statistically significant difference. 

## Results

### Patient characteristics

In this study 567 controls and 505 patients were recruited. Demographic and clinical characterizations of the study population are listed in Table 2(Tab. 2) and Table 3(Tab. 3). The mean age of the control and the patient group was 50.52±12.29 and 43.45±12.21, respectively (Table 2(Tab. 2)). The study of the demographic characteristics between patients and controls shows statistically significant differences in age, age of menarche (year), age of menopause (year) and age of first and last gestation. A significant association between cases and controls (P-*_value_*<0.05) was also found in age, age of menarche (year), age of menopause (year) and age of first and last gestation. Moreover, clinical characteristics of the target population presenting that most patients had invasive ductal carcinoma with ER^+^, PR^+^ and HER2^- ^ status (Table 3(Tab. 3)).

### Allele frequencies and association between SNPs and haplotypes with breast cancer susceptibility

All genotypes and allele frequencies in control samples were in HWE. More investigation revealed that there was a strong significant association between alleles and genotypes of *rs4784227-CASC16* with breast cancer risk (Table 4(Tab. 4)). In contrast, there was no significant statistical association between alleles and genotypes of *rs4782447-ACSF3* with the risk factors (Table 5(Tab. 5) and Table 6(Tab. 6)). We did not also find any association between alleles and genotypes of *rs4784227-CASC16* with the risk factors (Table 6(Tab. 6)). 

Furthermore, with two SNPs, we constructed four haplotypes (Table 7(Tab. 7)). A significant association was demonstrated between TA haplotype and breast cancer risk (Table 7(Tab. 7)). This haplotype results in decrease risk of breast cancers (OR=0.78; 95% CI (0.62-0.96); P*_-value_*=**0.025**). The association of haplotypes and risk factors were evaluated by crosstab program in SPSS 20. There was no association between haplotypes and the risk factors of breast cancer in all samples. 

## Discussion

In the present study we evaluated the association of two related SNPs in *16q *locus including *rs4784227-CASC16* and *rs4782447-ACSF3* and their haplotypes with the risk of breast cancer and risk factors in Iranian population. There was an association between *rs4784227-CASC16* with the risk of breast cancer. However, there was no association between *rs4784227-CASC16* and *rs4782447-ACSF3* and risk factors using different analysis models. Furthermore, there was a significant association between AT haplotype and risk of breast cancer that indicated the combination of haplotype and its effects may influence the risk of breast cancer in the populations. In other hand, the effect of the AT haplotype may be due to the more pronounced effect of *rs4784227-CASC16*.

In our study, consisting of 505 patients and 567 controls, genotype frequencies of *rs4784227-CASC16* were TT (14.9 % in cases and 10.5 % in controls); and CT (41.8 % in cases and 38.7 % in controls). There was a significant association between TT and CT genotypes with the risk of breast cancer. Furthermore, in our study, risk allele frequency (T allele) was 0.39 in patients. A similar case-control study in Iran represented significant association between CT-*rs4784227-CASC16* and the risk of breast cancer (60 % in 126 cases, 27.77 % in 160 controls). Additionally, in Iranian and Korean populations the risk allele frequency for T- *rs4784227-CASC16* allele was 0.26 and 0.24 to 0.29, respectively (Hajizadeh et al., 2017[13]; Kim et al., 2012[17]; Long et al., 2010[19]). The frequency of G-*rs4782447-ACSF3* allele as a risk allele in the present study was 0.38 in patients. There is no report for association of the *rs4782447-ACSF3* in Iranian population. 

It is suggested that TOX3 may be a risk factor for breast cancer development through pleotropic effects; TOX3 not only has a key role in tumorigenesis, but also might enhance the cell survival of especial tumour cells (Shan et al., 2013[27]). In this context, *16q12* SNPs and nearby regions are located in the introns of a non-protein coding gene. As such, it has been recommended that risky alleles may modulate gene expression by changing the enhancers activity (Abecasis et al., 2010[1]; Wasserman et al., 2010[34]). It indicates breast cancer *rs4782447-ACSF3* and *rs4784227-CASC16* SNPs are enhanced for FOXA1 DNA binding sequences and modification of the H3K4me1 histone (Cowper-Sal·lari et al., 2012[8]; Jia et al., 2009[15]; Meyer and Carroll, 2012[22]). The ability of FOXA1 to bind to DNA is crucial for opening of chromatin and nucleosome positioning sequences for recruitment of transcription factor (Cowper-Sal·lari et al., 2012[8]). Additionally, it disclosed this enrichment is factor-specific, cell-type-specific and specific types of cancer (Jia et al., 2009[15]). Thus, FOXA1 is associated with ER, and likely regulates the *TOX3 *promoter activity (Ross-Innes et al., 2012[24]). Researchers have shown that *rs4782447-ACSF3 *and* rs4784227-CASC16* may disrupt enhancer function by FOXA1-binding affinity-modulation therefore can change *TOX3* expression (Cowper-Sal·lari et al., 2012[8]; Meyer and Carroll, 2012[22]). 

The* rs4782447-ACSF3* SNP leads to the FOXA1 binding sequence change and consequently may increase the affinity of FOXA1 interacting to the *TOX3* gene promoter (Meyer and Carroll, 2012[22]). Furthermore, *G-rs4782447-ACSF3* slightly changes the binding sequence of FOXA1 and it is believed that it may enhance the DNA-binding affinity of FOXA1 (Figure 1(Fig. 1)). It has been shown that silencing expression of *TOX3* enhances cell proliferation *in vitro,* suggesting the effect of *rs4782447-ACSF3* on the expression of* TOX3 in vitro *(Meyer and Carroll, 2012[22]). Since that, Meyer and Carroll (2012[22]) suggested a tumour suppressor role for TOX3 in breast cancer. 

Another important SNP related to *TOX3* and FOXA1 is *rs4784227-CASC16* SNP, located 18.4 Kb upstream of the *TOX3 *gene (Cowper-Sal·lari et al., 2012[8]). Similarly, the statistically significant association was indicated between *rs4784227-CASC16* and risk of breast cancer among European, Southern China, and Korean populations (Easton et al., 2007[9]; He et al., 2014[14]; Kim et al., 2012[17]; Long et al., 2010[19]); moreover, consistent with our result, there was no report by these studies for an association with receptor status. The place for *rs4784227-CASC16* on FOXA1 genomic for interaction is on the eighth position of the FKH motif recognized *via* FOXA1 (Lupien et al., 2008[20]). In this regards, affinity DNA site for FOXA protein was enhanced for the *T-rs4784227-CASC16* compared with the C*-rs4784227-CASC16* (Katika and Hurtado, 2013[16]). It is suggested rs4784227-CASC16 modulates the chromatin affinity for FOXA1, exemplified by the *rs4784227-CASC16* effect on the promoter of the *TOX3 *gene identify (Cowper-Sal·lari et al., 2012[8]). It has also been shown that *T-rs4784227-CASC16* favours FOXA1-binding affinity over the C allele. Moreover, allele-specific directed ChIP assays indicated FOXA1 is modulated by the *T-rs4784227-CASC16 in vivo *(Lupien et al., 2008[20]). Interestingly, FOXA1 commonly stimulates gene expression, and co-binding to DNA sequence with Groucho (Gro)/transducin-like enhancer of split (TLE) proteins lead to local chromatin condensation and transcriptional repression (Wright et al., 2010[35]). The Gro/TLE protein, as co-repressors, do not directly connected to DNA sequence, but in contrast they are bound to the sequence of DNA through DNA-binding repressor proteins (Chen and Courey, 2000[7]). The risk variant *T-rs4784227-CASC16* associated with enhanced FOXA1 binding is strongly bound *via* Groucho/TLE versus the C allele. Additionally, H3K9Ac (a chromatin signature of active enhancers) is less observed at the *T-rs4784227-CASC16 *compared to the C allele (Cowper-Sal·lari et al., 2012[8]; Ernst et al., 2011[10]). It shows that the risk allele *T-rs4784227-CASC16* has led to a reduction in *TOX3 *gene expression because of an increase in the TLE repressor affinity recruitment that decreases the stability of the enhancer (Cowper-Sal·lari et al., 2012[8]). 

Additionally, *rs4784227-CASC16* has been associated with the expression of RB transcriptional corepressor like 2 (RBL2) protein as a regulatory sequence of the *RBL2* gene, and may also affect the risk of breast cancer (Udler et al., 2010[33]). In contrast, Cowper-Sal·lari et al. indicated that there is no association between *rs4784227-CASC16* and RBL2 in breast cancer cell lines (Cowper-Sal·lari et al., 2012[8]).

Collectively***, ***the expression of *TOX3* has been correlated with breast cancer and is important in revealing biological mechanisms, which makes a bridge between pathways and diseases. More functional researches may help increase our understanding of the exact biological features of breast cancer. 

## Notes

Zahra Farjami, Susan Darroudi and Seyed Hasan Ayati contributed equally as second authors.

Mohammad Mahdi Kushyar and Alireza Pasdar (Department of Medical Genetics, Faculty of Medicine, Mashhad University of Medical Sciences, Mashhad, Iran; E-mail: PasdarA@mums.ac.ir, a.pasdar@abdn.ac.uk) contributed equally as corresponding authors.

## Conflict of interest

The authors declare that they have no conflicts of interest.

## Figures and Tables

**Table 1:**
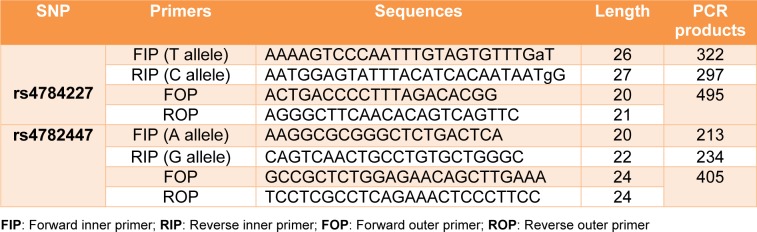
Primer sequences used for ARMS-PCR genotyping

**Table 2:**
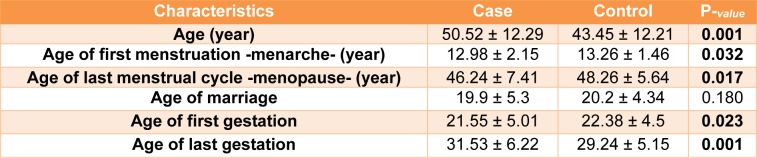
Controls and cases characteristics

**Table 3:**
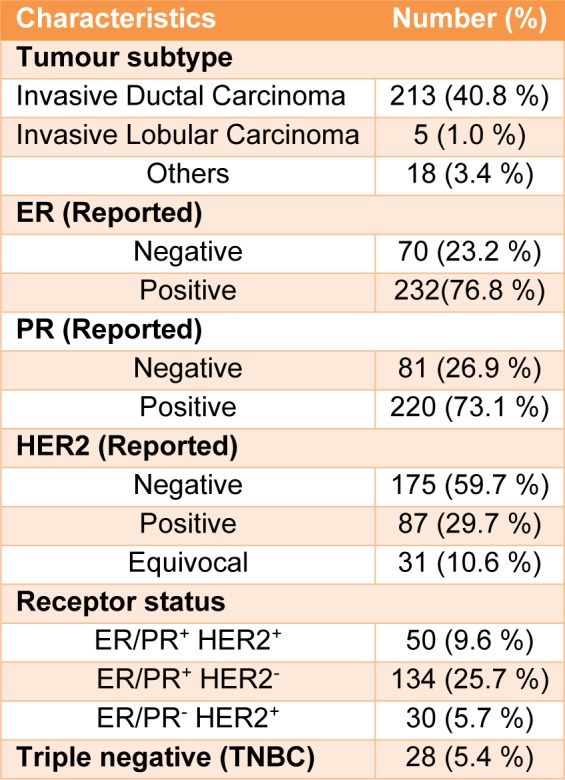
Frequency distribution of tumour characteristics of cases

**Table 4:**
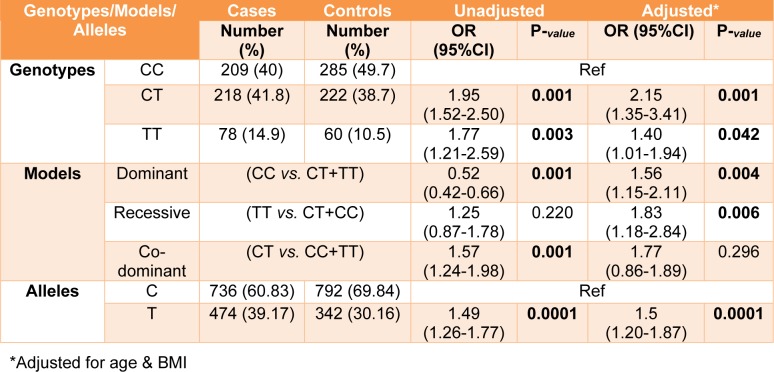
Distribution of genotypes and allele frequency of rs4784227 polymorphisms in breast cancer cases and controls

**Table 5:**
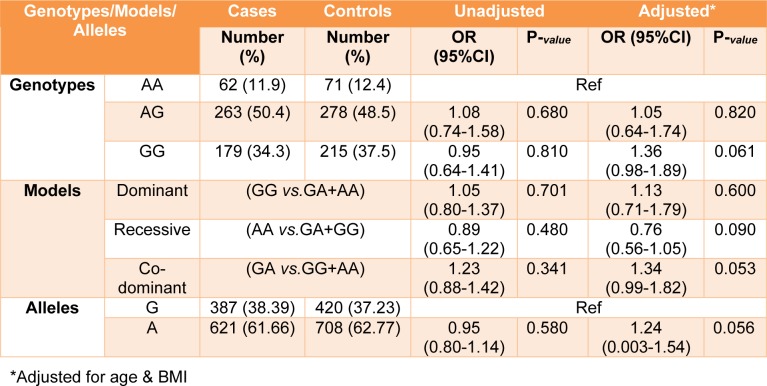
Distribution of genotypes and allele frequency of rs4782447 polymorphisms in breast cancer cases and controls

**Table 6:**
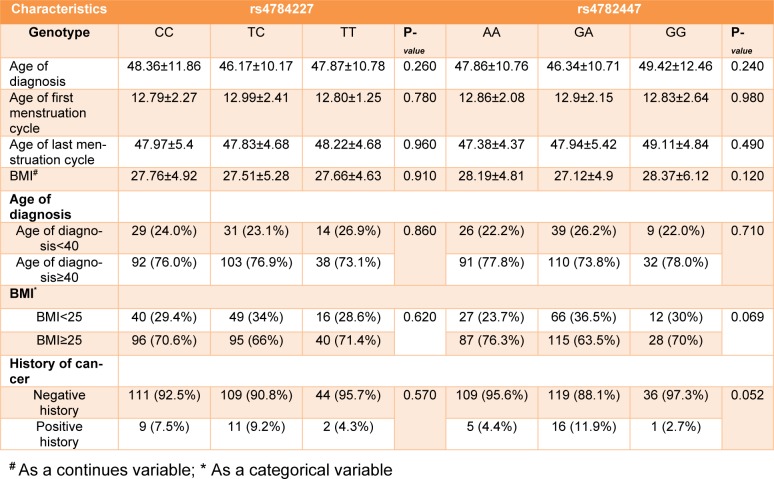
The association of rs4784227 and rs4782447 and demographic factors in cases

**Table 7:**
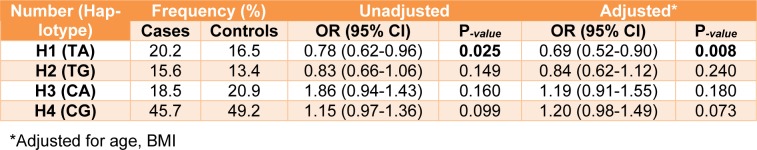
Haplotypes frequencies of case and controls

## References

[1] Abecasis GR, Altshuler D, Auton A, Brooks LD, Durbin RM, Gibbs RA (2010). A map of human genome variation from population-scale sequencing. Nature.

[2] Barrdahl M, Canzian F, Lindstrom S, Shui I, Black A, Hoover RN (2015). Association of breast cancer risk loci with breast cancer survival. Int J Cancer.

[3] Bernardo GM, Keri RA (2012). FOXA1: a transcription factor with parallel functions in development and cancer. Biosci Rep.

[4] Bernardo GM, Lozada KL, Miedler JD, Harburg G, Hewitt SC, Mosley JD (2010). FOXA1 is an essential determinant of ERα expression and mammary ductal morphogenesis. Development.

[5] Carroll JS, Liu XS, Brodsky AS, Li W, Meyer CA, Szary AJ (2005). Chromosome-wide mapping of estrogen receptor binding reveals long-range regulation requiring the forkhead protein FoxA1. Cell.

[6] Chalabi N, Bernard-Gallon DJ, Bignon YJ, Kwiatkowski F, Agier M, Vidal V (2008). Comparative clinical and transcriptomal profiles of breast cancer between French and South Mediterranean patients show minor but significative biological differences. Cancer Genom Proteom.

[7] Chen G, Courey AJ (2000). Groucho/TLE family proteins and transcriptional repression. Gene.

[8] Cowper-Sal lari R, Zhang X, Wright JB, Bailey SD, Cole MD, Eeckhoute J (2012). Breast cancer risk-associated SNPs modulate the affinity of chromatin for FOXA1 and alter gene expression. Nature Genet.

[9] Easton DF, Pooley KA, Dunning AM, Pharoah PD, Thompson D, Ballinger DG (2007). Genome-wide association study identifies novel breast cancer susceptibility loci. Nature.

[10] Ernst J, Kheradpour P, Mikkelsen TS, Shoresh N, Ward LD, Epstein CB (2011). Mapping and analysis of chromatin state dynamics in nine human cell types. Nature.

[11] Ghoussaini M, Pharoah PDP, Easton DF (2013). Inherited genetic susceptibility to breast cancer: the beginning of the end or the end of the beginning?. Am J Pathol.

[12] Gudmundsdottir ET, Barkardottir RB, Arason A, Gunnarsson H, Amundadottir LT, Agnarsson BA (2012). The risk allele of SNP rs3803662 and the mRNA level of its closest genes TOX3 and LOC643714 predict adverse outcome for breast cancer patients. BMC Cancer.

[13] Hajizadeh A, Houshmand M, Hosseini M (2017). TNP1 non-gene region and influences tumor characteristics by low-risk alleles in breast cancer. QUID: Investigación, Ciencia y Tecnología.

[14] He X, Yao G, Li F, Li M, Yang X (2014). Risk-association of five SNPs in TOX3/LOC643714 with breast cancer in southern China. Int J Mol Sci.

[15] Jia L, Landan G, Pomerantz M, Jaschek R, Herman P, Reich D (2009). Functional enhancers at the gene-poor 8q24 cancer-linked locus. PLOS Genetics.

[16] Katika MR, Hurtado A (2013). A functional link between FOXA1 and breast cancer SNPs. Breast Cancer Res.

[17] Kim HC, Lee JY, Sung H, Choi JY, Park SK, Lee KM (2012). A genome-wide association study identifies a breast cancer risk variant in ERBB4 at 2q34: Results from the Seoul Breast Cancer Study. Breast Cancer Res.

[18] Kong SL, Li G, Loh SL, Sung WK, Liu ET (2011). Cellular reprogramming by the conjoint action of ERalpha, FOXA1, and GATA3 to a ligand-inducible growth state. Mol Syst Biol.

[19] Long J, Cai Q, Shu XO, Qu S, Li C, Zheng Y (2010). Identification of a functional genetic variant at 16q12.1 for breast cancer risk: results from the Asia Breast Cancer Consortium. PLoS Genet.

[20] Lupien M, Eeckhoute J, Meyer CA, Wang Q, Zhang Y, Li W (2008). FoxA1 translates epigenetic signatures into enhancer-driven lineage-specific transcription. Cell.

[21] Mahfoudh W, Bouaouina N, Ahmed SB, Gabbouj S, Shan J, Mathew R (2012). Hereditary breast cancer in Middle Eastern and North African (MENA) populations: identification of novel, recurrent and founder BRCA1 mutations in the Tunisian population. Mol Biol Rep.

[22] Meyer KB, Carroll JS (2012). FOXA1 and breast cancer risk. Nature Genet.

[23] O'Flaherty E, Kaye J (2003). TOX defines a conserved subfamily of HMG-box proteins. BMC Genomics.

[24] Ross-Innes CS, Stark R, Teschendorff AE, Holmes KA, Ali HR, Dunning MJ (2012). Differential oestrogen receptor binding is associated with clinical outcome in breast cancer. Nature.

[25] Ruiz-Narvaez EA, Rosenberg L, Cozier YC, Cupples LA, Adams-Campbell LL, Palmer JR (2010). Polymorphisms in the TOX3/LOC643714 locus and risk of breast cancer in African-American women. Cancer Epidemiol Biomarkers Prev.

[26] Seksenyan A (2013). The role of nuclear factor TOX3 in mammary gland biology and breast cancer.

[27] Shan J, Dsouza SP, Bakhru S, Al-Azwani EK, Ascierto ML, Sastry KS (2013). TNRC9 downregulates BRCA1 expression and promotes breast cancer aggressiveness. Cancer Res.

[28] Stacey SN, Manolescu A, Sulem P, Rafnar T, Gudmundsson J, Gudjonsson SA (2007). Common variants on chromosomes 2q35 and 16q12 confer susceptibility to estrogen receptor-positive breast cancer. Nature Genet.

[29] Stephens M, Smith NJ, Donnelly P (2001). A new statistical method for haplotype reconstruction from population data. Am J Hum Genet.

[30] Tajbakhsh A, Afzal Javan F, Fazeli M, Rivandi M, Kushyar MM, Nassiri M (2017). TOX3 Gene polymorphisms and breast cancer;effects and implications of the variations: review article. Tehran Univ Med J.

[31] Tajbakhsh A, Javan FA, Rivandi M, Moezzi A, Abedini S, Asghari M (2019). Significant association of TOX3/LOC643714 locus-rs3803662 and breast cancer risk in a cohort of Iranian population. Mol Biol Rep.

[32] The Encode Project Consortium, Dunham I, Kundaje A, Aldred SF, Collins PJ, Davis CA, (2012). An integrated encyclopedia of DNA elements in the human genome. Nature.

[33] Udler MS, Ahmed S, Healey CS, Meyer K, Struewing J, Maranian M (2010). Fine scale mapping of the breast cancer 16q12 locus. Hum Mol Genet.

[34] Wasserman NF, Aneas I, Nobrega MA (2010). An 8q24 gene desert variant associated with prostate cancer risk confers differential in vivo activity to a MYC enhancer. Genome Res.

[35] Wright JB, Brown SJ, Cole MD (2010). Upregulation of c-MYC in cis through a large chromatin loop linked to a cancer risk-associated single-nucleotide polymorphism in colorectal cancer cells. Mol Cell Biol.

[36] Yoo J, Lee Y, Kim Y, Rha SY, Kim Y (2008). SNP Analyzer 2.0: A web-based integrated workbench for linkage disequilibrium analysis and association analysis. BMC Bioinformatics.

